# External validation of predictive equations for determining the need for in-flight oxygen supplementation in patients with respiratory disease

**DOI:** 10.3389/fphys.2026.1946975

**Published:** 2026-09-17

**Authors:** Murielle Emma Beaupré, Gao Sheng Huang, Bruno-Pierre Dubé

**Affiliations:** 1 Service de Pneumologie, Centre Hospitalier de l’Université de Montréal (CHUM), Université de Montréal, Montreal, QC, Canada; 2 Centre de Recherche du Centre Hospitalier de l’Université de Montréal (CRCHUM), Montreal, QC, Canada

**Keywords:** air travel, desaturation, fitness to fly, hypoxic challenge, in-flight oxygen, predictive equations

## Abstract

**Rationale:**

Patients with chronic respiratory diseases may develop clinically significant hypoxemia during commercial air travel because of reduced cabin pressure. In-flight oxygen prescription is typically guided by hypoxic challenge testing (HCT), a procedure that is time-consuming, costly, and not universally available. Several predictive equations based on sea-level physiological measurements have been proposed as alternatives. We aimed to externally validate six published predictive equations by comparing their performance with HCT in a large, heterogeneous cohort of patients with chronic respiratory diseases.

**Methods:**

We retrospectively analyzed all patients who underwent HCT at our center between 2016 and 2026. Sea-level arterial blood gases, pulmonary function tests, and relevant clinical data were collected. Six published predictive equations (E1–E6) were used to estimate in-flight PaO_2_ and compared with PaO_2_ measured during HCT.

**Results:**

A total of 177 patients were included: 39 (22%) with COPD, 64 (36%) with ILD, 34 (19%) with cystic fibrosis, and 40 (23%) with other chronic respiratory diseases. Significant correlations between predicted in-flight PaO_2_ and HCT PaO_2_ were observed (ρ = 0.48 for E1 and E2 to 0.84 for E5; all *p* ≤ 0.001), but diagnostic performance remained limited. Across all patients, predictive equations demonstrated high specificity (76% for E2 to 98% for E6) but inadequate sensitivity (11% for E6 to 67% for E2) for identifying patients requiring supplemental oxygen. Cohen’s κ coefficients ranged from 0.07 (95% CI, −0.06 to 0.20) for E4 to 0.43 (95% CI, 0.27–0.59) for E2. Areas under the ROC curve ranged from 0.71 (95% CI, 0.62–0.81) for E4 to 0.79 (95% CI, 0.70–0.88) for E1.

**Conclusions:**

In this large external validation study, currently available predictive equations demonstrated insufficient diagnostic performance to safely replace HCT for determining the need for in-flight oxygen supplementation. Although several equations showed moderate to strong agreement with measured HCT PaO_2_, inadequate sensitivity resulted in clinically important misclassification of patients requiring supplemental oxygen. HCT should remain the reference standard for evaluating patients in whom the need for in-flight oxygen supplementation is uncertain.

## 1 Introduction

Patients with chronic respiratory disease are at increased risk of hypoxemia during commercial air travel because aircraft cabins are pressurized to the equivalent of a maximum altitude of approximately 8,000 feet, resulting in a reduction in inspired oxygen tension. While healthy individuals generally maintain adequate oxygenation under these conditions, patients with impaired pulmonary function may experience clinically significant desaturation. Current British Thoracic Society guidelines recommend hypoxic challenge testing (HCT) as the reference standard for assessing the need for in-flight oxygen supplementation in patients considered at risk. However, HCT is time-consuming, costly, and not universally available, limiting its accessibility in routine clinical practice (Coker et al., 2022).

Several predictive equations based on sea-level arterial blood gases and pulmonary function measurements have been developed as potential alternatives to HCT. Early equations proposed by Gong et al. (1984) and Dillard et al. (1989) were derived from small cohorts of patients with chronic obstructive pulmonary disease (COPD) and subsequently evaluated in external validation studies. Although these equations are simple to apply, their reported diagnostic performance has been inconsistent, with studies demonstrating poor agreement with HCT and limited clinical accuracy. Moreover, patients with similar resting oxygenation and pulmonary function may exhibit markedly different responses to hypoxic exposure, highlighting the limitations of relying solely on baseline physiological measurements. Identifying patients who could safely avoid HCT has therefore remained a clinical challenge (Marchand, 2008).

More recently, Billings et al. (2013) developed non-linear predictive models incorporating additional clinical variables, which demonstrated improved performance compared with earlier linear equations. Although these models appeared promising, they require external validation in independent patient populations before they can be recommended for routine clinical use. Furthermore, most predictive equations have been developed from relatively small cohorts consisting primarily of patients with COPD, and their performance in broader populations with diverse respiratory diseases remains uncertain (Martin et al., 2007; Robson et al., 2008; Bradi et al., 2009).

The objective of the present study was therefore to evaluate the performance of previously published predictive equations for estimating in-flight oxygenation in a large, real-world cohort of patients undergoing HCT. Specifically, we compared the agreement, predictive accuracy, and diagnostic performance of six published equations against measured HCT results across patients with COPD, interstitial lung disease, cystic fibrosis, and other respiratory disorders.

## 2 Methods

### 2.1 Study design and population

This retrospective observational study included consecutive adult patients who underwent hypoxic challenge testing (HCT) at the respiratory function laboratory of the Centre Hospitalier de l’Université de Montréal (CHUM) between January 2016 and February 2026. The study was approved by the institutional research ethics board (number 20.260). Because of the retrospective nature of this study and the strictly anonymous collection of data, no consent was necessary. Patients less than 18 years of age were excluded. Patients were classified according to their primary respiratory diagnosis into COPD, ILD, cystic fibrosis (CF), or other respiratory diseases.

Demographic and clinical variables extracted from electronic medical records included age, sex, anthropometric measurements, smoking history, cumulative tobacco exposure (pack-years), and modified Medical Research Council (mMRC) dyspnea score.

The pulmonary function test performed closest to the HCT was used for analysis, with measures including forced expiratory volume in one second (FEV_1_), forced vital capacity (FVC), residual volume (RV), thoracic gas volume (TGV), total lung capacity (TLC) and diffusion capacity (D_L_CO) when available. All tests were performed according to ATS guidelines (Graham et al., 2017; Graham et al., 2019; Stanojevic et al., 2022; Bhakta et al., 2023).

Resting arterial blood gases (ABGs) were obtained immediately before HCT in all patients except those with CF, for whom institutional practice relies on pulse oximetry rather than arterial sampling before testing. At the end of the HCT, arterial blood gases (or pulse oximetry in patients with CF) were repeated and recorded for analysis.

### 2.2 Hypoxic challenge testing

Hypoxic challenge testing was performed according to current guidelines. Patients breathed a 15% oxygen gas mixture through a sealed facemask for 20 minutes while pulse oximetry and clinical symptoms were monitored throughout the procedure. Arterial blood gas was performed immediately after HCT to determine simulated in-flight PaO2 (PaO2-HCT). HCT was terminated early if SpO2 fell below 85%.

A positive HCT was defined as a PaO_2_ ≤ 50 mmHg (or a SpO_2_ ≤ 85% for patients with CF).

### 2.3 Predictive equations

Six predictive equations (E1-E6) were used to estimate altitude PaO_2_ using sea-level measurements.

1. Equation 1, Billings et al. (2013):

(1) $$\text{PaO}2\text{ Alt}=(0.559496\times [\text{PaO}2\text{RA}])-(0.001319\times [\text{PaO}2\text{RA}]2)-(0.000189\times {\left[\text{PaCO}2\text{RA}\right]}^{3}+(0.799106\times [\text{PaCO}2\text{RA}])$$

2. Equation 2, Dillard et al. (1989):

(2) PaO2 Alt=0.410 x PaO2RA+17.652

3. Equation 3, Dillard et al. (1989):

(3) PaO2 Alt=0.519 x PaO2RA+11.855 x FEV1 (L)−1.760

4. Equation 4, Dillard et al. (1989):

(4) PaO2 Alt=0.453 x PaO2RA+0.386 x (FEV1%)+2.44

5. Equation 5, Gong et al. (1984):

(5) PaO2 Alt=22.8−(2.74 x altitude in thousands of feet)+0.68 x PaO2RA

6. Equation 6, Billings et al. (2013):

(6) SaO2 % Alt=(0.933164 x [SaO2RA%])

E1 and E6 were derived from the non-linear models described by Billings et al. (2013), whereas E2–4 originated from the linear equations developed by Dillard et al. (1989). E5 was derived from the equation proposed by Gong et al. (1984). Because arterial blood gases were unavailable in patients with CF, E6, which predicts arterial oxygen saturation, was evaluated in this subgroup.

In the equations detailed above, PaO_2_Alt = partial pressure of oxygen at altitude, PaO_2_RA = partial pressure of oxygen on room air, at sea-level, PaCO_2_RA = partial pressure of carbon dioxide on room air, at sea-level, FEV_1_ (L) = forced expiratory volume in one second, in liters and FEV_1_% represents forced expiratory volume in one second, in percentage of predicted value.

### 2.4 Outcomes

The primary outcome was the agreement between predicted and measured in-flight oxygenation during HCT.

Secondary outcomes included diagnostic performance of each predictive equation across disease diagnostic categories.

### 2.5 Statistical analysis

The performance of the predictive equations was evaluated by comparing predicted in-flight PaO_2_ or SaO_2_ values with observed values obtained during formal hypoxic challenge testing. Correlations between predicted and measured values were assessed using Spearman correlation coefficients.

Agreement between predicted and measured values was assessed using Bland-Altman analyses. For each equation, mean bias was calculated as the difference between predicted and observed values (predicted – observed), and the root mean square error (RMSE) and mean absolute error were calculated to quantify predictive accuracy.

The diagnostic performance of each equation for predicting a positive HCT result was evaluated by calculating sensitivity, specificity, positive predictive value (PPV), and negative predictive value (NPV). Agreement between the binary classification provided by each equation and the observed HCT outcome was assessed using Cohen’s kappa coefficient (κ) with corresponding 95% confidence intervals.

Receiver operating characteristic (ROC) curves were created for each equation using HCT positivity as the reference standard. Areas under the ROC curves (AUCs) with 95% confidence intervals were calculated. In addition to evaluating diagnostic performance at the established clinical threshold, optimal threshold values were determined using the highest combined sensitivity and specificity to assess whether alternative decision thresholds improved classification performance in this external cohort.

To further assess the clinical utility of the equations as rule-out or screening tools, negative predictive value (NPV) was evaluated at progressively higher predicted oxygenation thresholds. For E1–E5, NPV was calculated at predicted PaO_2_ thresholds of 50, 55, 60, and 65 mmHg, while E6 was evaluated at predicted SaO_2_ thresholds of 85%, 90%, and 92%. For each threshold, the proportion of eligible patients with predicted values above the threshold was reported.

Subgroup analyses were performed according to underlying diagnosis. Because arterial blood gas measurements during HCT were unavailable in patients with cystic fibrosis (CF), comparisons of PaO_2_-based equations were restricted to patients with chronic obstructive pulmonary disease (COPD) and interstitial lung disease (ILD). Differences in absolute prediction errors between COPD and ILD were compared using independent-samples t-tests.

A two-sided p-value <0.05 was considered statistically significant. Statistical analyses were performed using IBM SPSS Statistics Version 29.0.2.0 (IBM Corp., Armonk, NY, USA).

## 3 Results

### 3.1 Study population

A total of 177 consecutive patients who underwent HCT were included (39 COPD, 64 ILD, 34 CF, and 40 with other respiratory diseases). Among patients in the “other” groups, underlying diagnosis included: lung cancer, severe obstructive sleep apnea, diaphragmatic weakness and bronchiectasis among other miscellaneous respiratory diseases. E1, E2, and E5 could be calculated in 123 patients, whereas E3 and E4 could be calculated in 110 patients because FEV_1_ measurements were unavailable in 13 patients. Baseline characteristics of patients are detailed in **Table 1**.

**Table 1:** Baseline characteristics of patients.

		COPD N = 39	ILD N = 64	CF N = 34	Other N = 40	All N = 177
Age		69 ± 10	69 ± 9	38 ± 11	60 ± 18	61 ± 17
Sex N (%)	Male	25 (64.1%)	44 (68.8%)	18 (50.0%)	18 (45.0%)	105 (59.3%)
	Female	14 (35.9%)	20 (31.3%)	16 (50.0%)	22 (55.0%)	72 (40.7%)
Height (cm)		164.5 ± 8.7	166.1 ± 8.4	165.7 ± 9.7	163.0 ± 8.8	165.0 ± 8.8
Weight (kg)		71.7 ± 16.3	77.0 ± 15.8	64.9 ± 14.6	79.4 ± 23.3	74.1 ± 18.3
Smoking status N (%)	Never	8 (21.6%)	17 (27.0%)	33 (100.0%)	17 (54.8%)	75 (45.7%)
	Active	2 (5.4%)	2 (3.2%)	0 (0%)	2 (6.5%)	6 (3.7%)
	Former	27 (73.0%)	44 (69.8%)	0 (0%)	12 (38.7%)	83 (50.6%)
Pack-years		29 ± 24	23 ± 26	0 ± 0	13 ± 29	16 ± 26
mMRC N (%)	0	1 (2.9%)	0 (0%)	0 (0%)	1 (3.3%)	2 (1.3%)
	1	10 (29.4%)	6 (10.7%)	19 (61.3%)	16 (53.3%)	51 (33.8%)
	2	14 (41.2%)	29 (51.8%)	10 (32.3%)	7 (23.3%)	60(49.7%)
	3	6 (17.6%)	17 (30.4%)	1 (3.2%)	4 (13.3%)	28 (18.5%)
	4	3 (8.8%)	4 (7.1%)	1 (3.2%)	2 (6.7%)	10 (6.6%)
Sea level SpO2		95.89 ± 2.58	95.9 ± 2.5	96.1 ± 2.1	96.8 ± 2.2	96.1 ± 2.4
Sea level PaO2		79.0 ± 14.1	81.8 ± 13.7	–	84.0 ± 14,8	81.6 ± 14.1
Sea level SaO2		95.3 ± 2.4	95.6 ± 2.2	–	95.9 ± 2.9	95.6 ± 2.5
Sea level PCO2		38.9 ± 5.4	37.9 ± 4.0	–	40.2 ± 6.4	38.8 ± 5.1
Sea level HCO3		25.0 ± 3.0	24.4 ± 2.3	–	25.4 ± 2.6	24.8 ± 2.6
FEV1 (L)		1.4 ± 0.8	2.0 ± 0.6	1.4 ± 0.4	1.9 ± 0.8	1.7 ± 0.7
FEV1 (%predicted)		49.8 ± 22.5	76.01± 17.5	41.1 ± 10.0	72.2 ± 27.8	62.7 ± 24.5
FVC (Value)		2.8 ± 1.2	2.5 ± 0.8	2.9 ± 0.8	2.45 ± 1.0	2.6 ± 0.9
FVC (%predicted)		77.3 ± 22.6	70.3 ± 18.9	67.6 ± 11.8	72.0 ± 22.8	71.4 ± 19.4
Tiffeneau		49.6 ± 16.7	83.6 ± 7.9	50.6 ± 10.7	77.9 ± 12.4	68.8 ± 19.5
TLC (L)		6.2 ± 1.6	4.1 ± 01.1	6.2 ± 1.4	4.4 ± 1.1	5.2 ± 1.6
TLC (%predicted)		106.9 ± 21.4	70.8 ± 13.3	108.8 ± 14.0	81.3 ± 16.4	91.8 ± 23.1
DLCO (L)		13.7 ± 5.8	11.0 ± 3.8	21.4 ± 5.7	17.7 ± 6.2	14.8 ± 6.4
DLCO (%predicted)		58.2 ± 18.9	49.0 ± 15.0	81.1 ± 16.8	80.9 ± 21.2	63.0 ± 22.7

Overall, 72 patients (41%) had a positive HCT.

Patients with ILD demonstrated the lowest HCT PaO_2_ values, whereas patients with CF generally maintained higher oxygenation (**Figure 1**).

**Figure 1:**
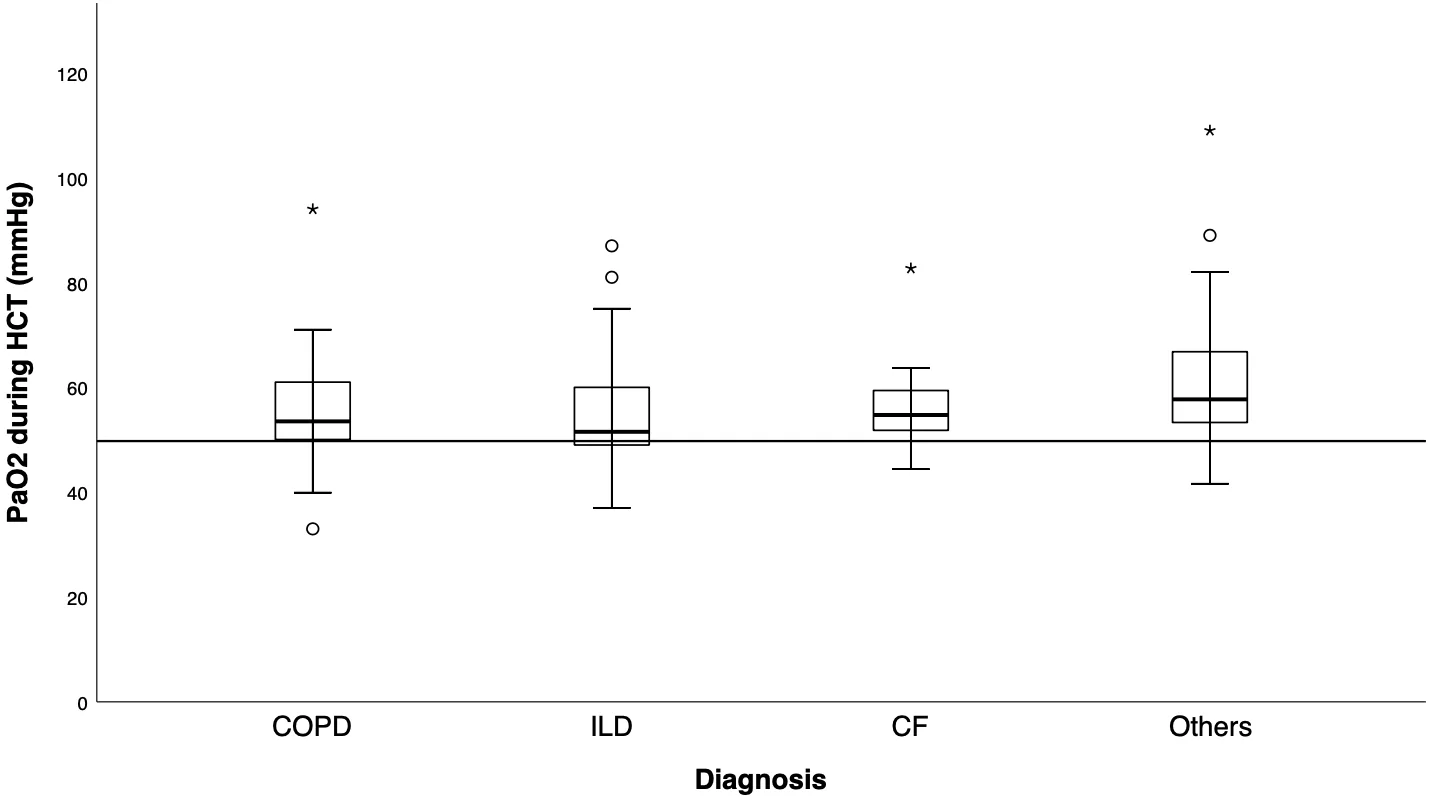
HCT-PaO2 results according to diagnostic category. * represents an extreme outlier in data.

### 3.2 Correlation between predicted and measured hypoxic values

All equations demonstrated statistically significant correlations with measured HCT values (all p<0.001), although the strength of correlation varied considerably. E5 showed the strongest correlation with measured PaO_2_ (ρ=0.84), whereas E1 and E2 demonstrated only moderate correlations (ρ=0.48). E6 demonstrated a moderate correlation with HCT SaO_2_ in patients with CF (ρ=0.51). (**Table 2**, **Figure 2**).

**Table 2:** Correlation, agreement, and predictive accuracy of equations for predicting in-flight hypoxemia.

	Correlation Spearman r	Mean bias (mmHg)	RMSE (mmHg)	Absolute error (mmHg)	κ (95%CI)
E1	0.48, p<0.001	-0.46 ± 10.1	10.1	6.0 ± 7.4	0.17 (0.05-0.29)
E2	0.48, p<0.001	-5.3 ± 10.1	10.2	7.4 ± 8.6	0.43 (0.27-0.59)
E3	0.51, p<0.001	6.4 ± 11.5	8.7	10.3 ± 8.2	0.24 (0.08-0.40)
E4	0.53, p<0.001	10.0 ± 10.9	8.5	12.1 ± 8.6	0.07 (-0.06-0.20)
E5	0.84, p<0.001	0.13 ± 10.9	10.1	7.4 ± 7.9	0.38 (0.23-0.53)
E6*	0.51, p<0.001	1.2 ± 4.5	4.5	3.6 ± 2.8	0.10 (0.01-0.19)

*For E6, mean bias, RMSE and absolute errors are reported for SaO_2_.

RMSE, root mean square error; SD, standard deviation; κ, Cohen’s kappa.

**Figure 2:**
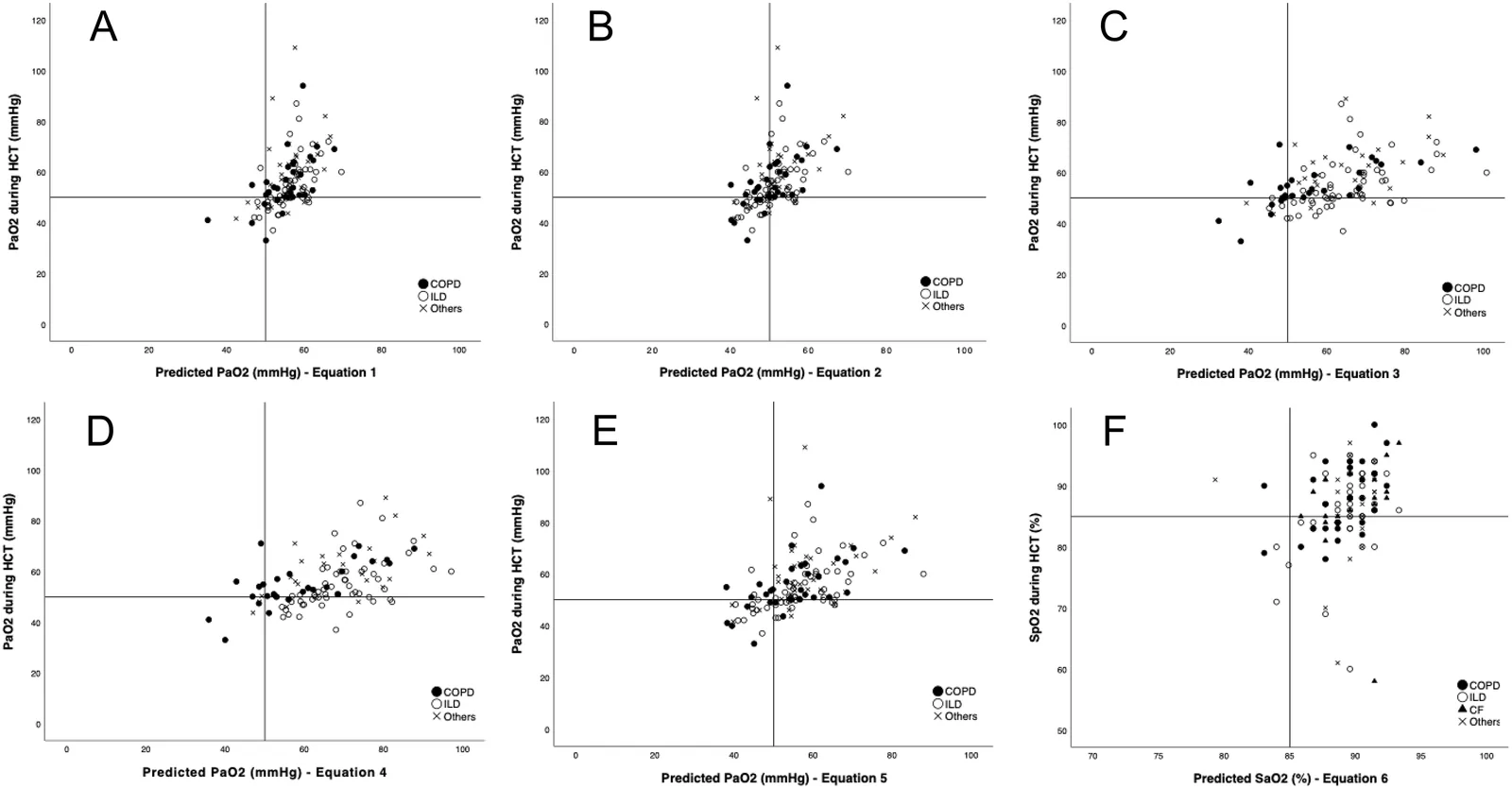
Correlation between measured and predicted PaO2 at altitude (SpO2 for Equation 6) for each predictive equation. **(A-F)** correspond to Equations 1-6. The number of observations differs between panels according to the availability of variables required for calculation of each equation.

### 3.3 Agreement between predicted and measured values

Mean bias varied substantially among the equations (**Table 2**, **Figure 3**). Agreement between predicted and measured PaO_2_ varied substantially among equations. E1 and E5 demonstrated minimal systematic bias, whereas E3 and E4 consistently overestimated altitude PaO_2_. Although RMSE values were broadly similar across equations, E1 demonstrated the smallest absolute prediction error.

**Figure 3:**
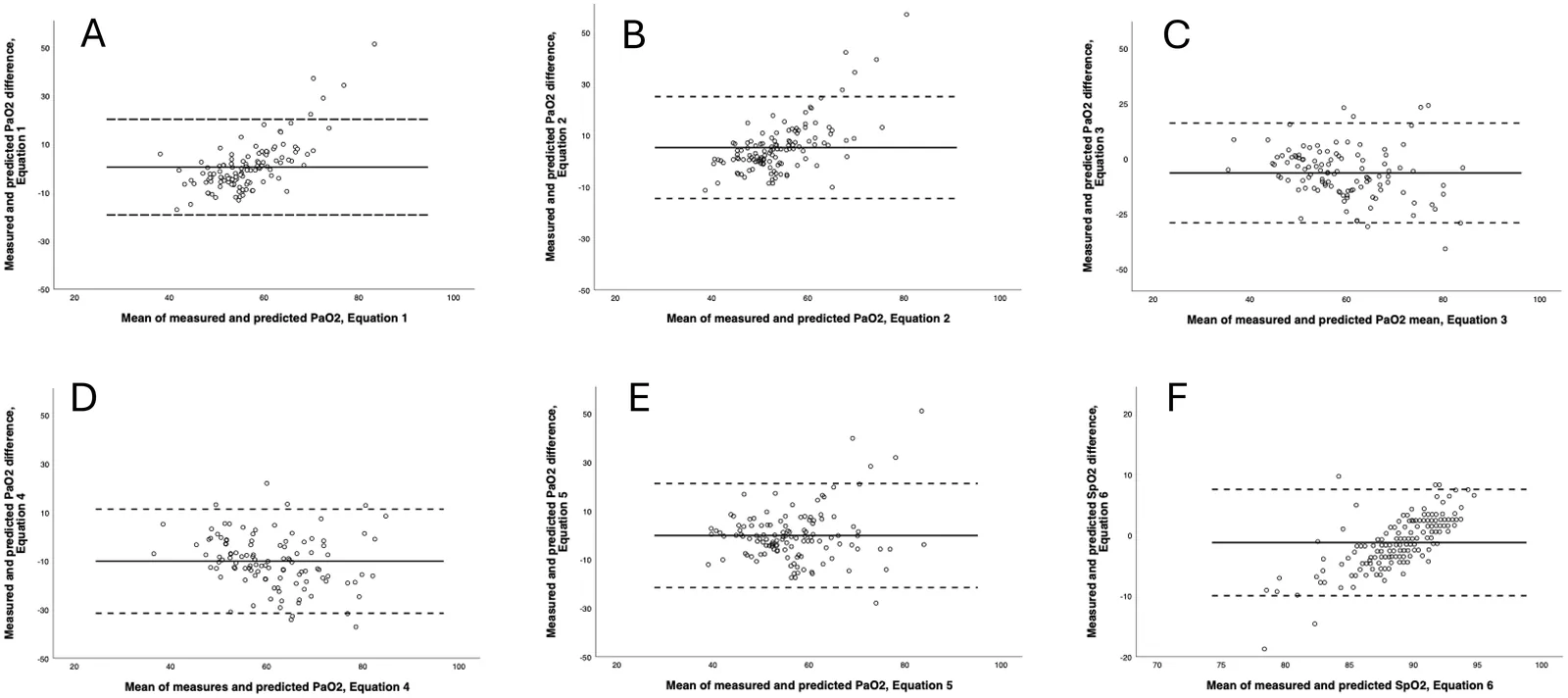
Bland-Altman plots for each predictive equation. Dotted lines represent the 95% limits of agreement of measured and observed values, and the solid line is their mean difference.

### 3.4 Diagnostic performance for predicting a positive HCT

The diagnostic performance of the predictive equations is summarized in **Table 3**. Diagnostic performance varied considerably between equations. Although specificity was consistently high (76–98%), sensitivity remained poor, ranging from only 11% to 67%. E2 demonstrated the highest sensitivity (67%), whereas E1 and E6 identified fewer than one in five patients with a positive HCT.

**Table 3:** Diagnostic performance of predictive equations for predicting a positive hypoxic challenge test.

	Sensitivity (%)	Specificity (%)	PPV (%)	NPV (%)
E1	19	97	83	62
E2	67	76	67	75
E3	32	91	73	63
E4	14	92	58	58
E5	44	92	80	69
E6	11	98	78	61

PPV, positive predictive value; NPV, negative predictive value.

### 3.5 Agreement of binary classification

Agreement between predicted and observed HCT classifications was generally poor (**Table 2**). E2 demonstrated the highest agreement (κ=0.43), followed by E5 (κ=0.38), whereas the remaining equations showed only slight-to-fair agreement.

### 3.6 Receiver operating characteristic analyses

All equations demonstrated significant discriminatory ability for identifying patients with a positive HCT, with AUC ranging from 0.71 to 0.79 (all p<0.001) (**Table 4**, **Figure 4**). E1 achieved the highest AUC (0.79; 95% CI, 0.70-0.88), although none of the equations demonstrated excellent discrimination.

**Table 4:** ROC analyses and optimal threshold values of predictive equations.

	AUC	p-value	95% CI	Best threshold	Sensitivity of threshold value	Specificity of threshold value
E1	0.79	<0.001	0.70-0.88	56	76%	75%
E2	0.77	<0.001	0.68-0.86	51	80%	66%
E3	0.73	<0.001	0.63-0.82	67	84%	51%
E4	0.71	<0.001	0.62-0.81	64	66%	72%
E5	0.77	<0.001	0.68-0.86	55	74%	75%
E6*	0.75	<0.001	0.67-0.82	89	62%	78%

For E6, threshold value is reported for SpO_2_.

AUC, area under the receiver operating characteristics curve.

**Figure 4:**
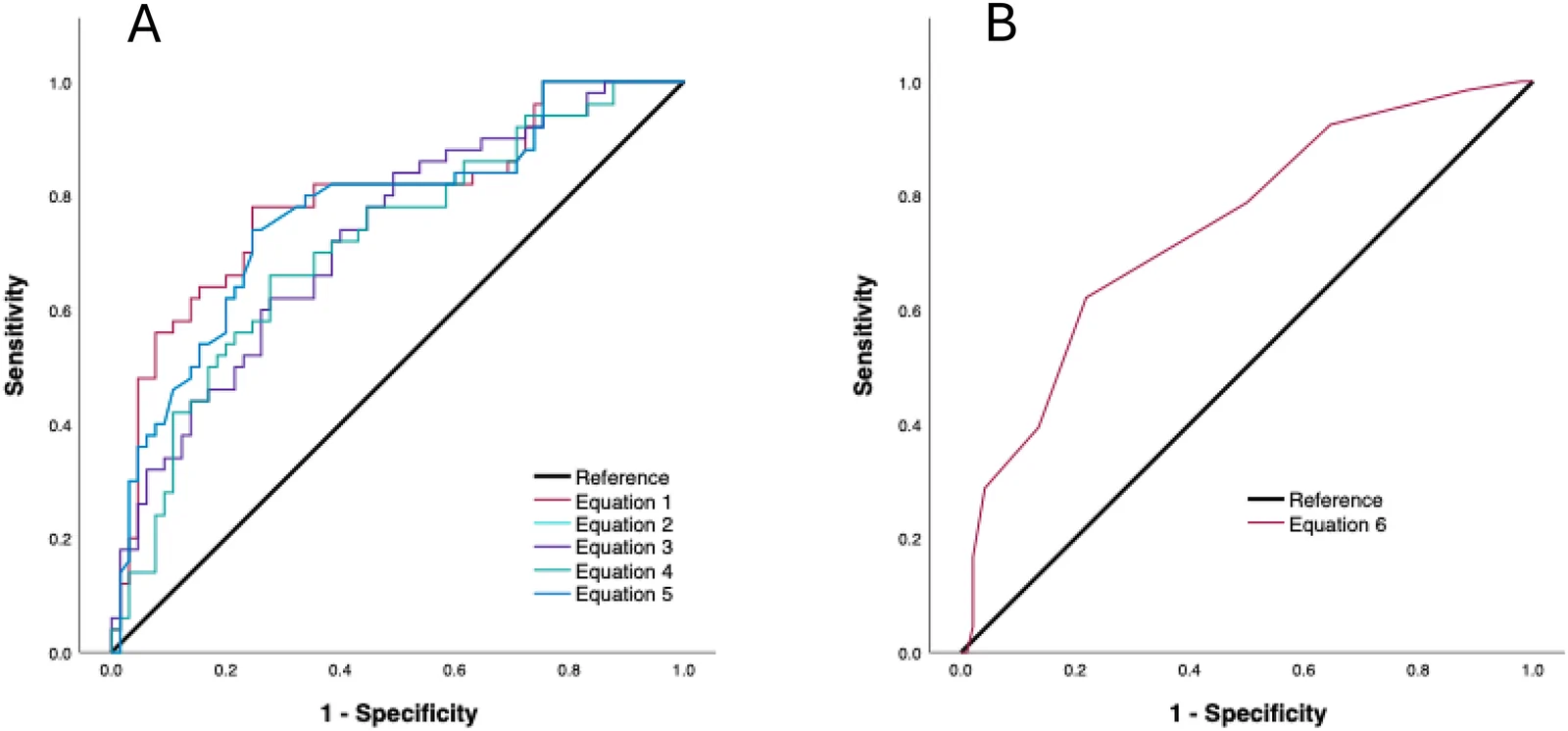
Receiver operating characteristic curves of the six predictive equations for their ability to predict a positive HCT test. **(A)** Equations 1–5, **(B)** Equation 6.

Optimal threshold values calculated from the ROC analyses varied widely, from 51 to 67 mmHg for the PaO_2_-based equations and 89% for E6. The optimal threshold identified for E2 was associated with the highest sensitivity (80%) among the clinically relevant thresholds.

Taken together, these findings indicate that good correlation with measured HCT values did not consistently translate into clinically useful classification of patients requiring supplemental oxygen.

### 3.7 Threshold analysis of optimal negative predictive value

Evaluation of progressively higher predicted oxygenation thresholds did not identify a consistently reliable rule-out threshold. Although NPV generally increased at higher predicted PaO_2_ values, this was accompanied by a marked reduction in the proportion of patients remaining above the threshold. For example, E2 reached an NPV of 100% at predicted PaO_2_ ≥60 mmHg, but only 5.4% of eligible patients met this criterion. Detailed results are presented in **Tables 1**–**4** of **Supplementary Materials**.

### 3.8 Performance according to underlying diagnosis

Prediction errors for E3 and E4 were significantly greater in ILD than COPD, whereas the remaining equations performed similarly in both disease groups (**Table 5**).

**Table 5:** Comparison of absolute prediction errors according to underlying diagnosis.

	COPD absolute error (mmHg)	ILD absolute error (mmHg)	CF absolute error (SaO_2)_	p-value
E1	6.3 ± 6.3	6.3 ± 5.7	–	0.96
E2	7.2 ± 1.3	5.9 ± 6.7	–	0.38
E3	7.6 ± 7.3	12.2 ± 8.4	–	0.01
E4	8.2 ± 6.0	15.1 ± 8.6	–	<0.001
E5	7.0 ± 6.6	6.8 ± 6.7	–	0.89
E6*	3.3 ± 2.3	3.6 ± 3.1	3.6 ± 2.3	0.72

*For E6, absolute errors are reported for SpO_2_.

ROC analyses showed comparable discriminatory performance for all equations, with overlapping AUC estimates across diagnostic groups (**Table 6**).

**Table 6:** Receiver operating characteristic analyses according to underlying diagnosis.

	COPD AUC (95% CI)	ILD AUC (95% CI)	CF AUC (95% CI)
E1	0.78 (0.61-0.95)	0.80 (0.68-0.92)	–
E2	0.77 (0.59-0.94)	0.79 (0.67-0.91)	–
E3	0.82 (0.66-0.97)	0.75 (0.63-0.88)	–
E4	0.80 (0.64-0.96)	0.77 (0.65-0.89)	–
E5	0.77 (0.59-0.94)	0.79 (0.67-0.91)	–
E6	0.71 (0.54-0.89)	0.78 (0.66-0.90)	0.73 (0.54-0.92)

## 4 Discussion

In this external validation study of six previously published predictive equations, none demonstrated sufficient diagnostic performance to safely replace hypoxic challenge testing for determining the need for in-flight oxygen supplementation. Although several equations correlated reasonably well with measured HCT values, their ability to identify patients requiring supplemental oxygen remained limited, primarily because of inadequate sensitivity. These findings suggest that accurate prediction of in-flight PaO_2_ does not necessarily translate into reliable clinical decision-making. To our knowledge, this is one of the largest studies to externally validate published predictive equations across multiple chronic respiratory diseases using formal HCT as the reference standard.

Although several equations showed moderate to strong correlations with measured HCT values, predicting in-flight hypoxemia from resting physiological measurements remains inherently challenging. Most published equations rely on a limited number of variables, such as resting PaO_2_, SpO_2_, FEV_1_, or PaCO_2_, which possibly cannot fully capture the complex physiological response to hypobaric hypoxia. Factors including ventilation-perfusion mismatch, diffusion impairment, pulmonary vascular abnormalities and the individual variability in the hypoxic ventilatory response may contribute differently to altitude-induced hypoxemia and are not entirely reflected by these baseline measurements. Furthermore, the equations evaluated in this study were largely derived from small cohorts of patients with COPD and may therefore be less applicable to more heterogeneous populations, such as those with ILD or CF. Importantly, even small prediction errors may result in clinically relevant misclassification as decisions regarding in-flight oxygen supplementation are based on fixed threshold values rather than continuous PaO_2_ measurements. These physiological and methodological limitations likely explain why the reasonable correlations with measured HCT values did not translate into sufficient diagnostic accuracy for clinical decision-making (Georges et al., 2021).

Among the evaluated models, the Gong equation (Gong et al., 1984) demonstrated the strongest correlation with measured HCT PaO_2_ and minimal systematic bias, indicating good performance for estimating altitude oxygenation at the population level. However, this did not translate into superior diagnostic accuracy for identifying patients requiring supplemental oxygen. This finding underscores an important distinction between predictive accuracy and clinical utility, as even relatively small prediction errors may shift patients across the treatment threshold, thereby reducing sensitivity despite good overall agreement. Similarly, although E2 showed the highest sensitivity and the strongest agreement with HCT classification, its sensitivity remained well below what can generally be considered acceptable for a screening tool intended to exclude the need for supplemental oxygen.

For disease-specific analyses, overall discriminatory performance was comparable between COPD and ILD, with overlapping AUC across diagnostic groups. However, prediction errors were significantly greater in ILD for E3 and E4. These differences may reflect the physiological characteristics captured by each individual equation. E3 and E4 were originally derived in patients with COPD and incorporate FEV_1_ as a predictor, whereas hypoxemia in ILD may be more strongly affected by diffusion capacity. Indeed, mechanisms such as diffusion capacity and ventilation-perfusion mismatch are not adequately reflected by spirometry. In contrast, prediction errors for E1, E2, and E5 did not differ significantly between COPD and ILD, suggesting that reduced performance in ILD is not uniform across predictive models. These findings highlight the importance of considering both equation structure and underlying respiratory physiology when extrapolating prediction models across heterogeneous disease populations. More recently, Robertson et al. (2025) reported that a resting PaO_2_>9.30 kPa (approximatively 70 mmHg) could identify patients with ILD likely to pass HCT, suggesting that disease-specific rule-out strategies may warrant further evaluation and external validation. Disease-specific interpretation in CF was more limited because arterial blood gases were not routinely obtained during HCT in this subgroup, precluding direct evaluation of the PaO_2_-based equations.

Furthermore, evaluation of the equations as potential screening tools did not identify a predicted PaO_2_ threshold that could reliably rule out the need for HCT while remaining applicable to a clinically meaningful proportion of patients. Although higher thresholds generally increased NPV, this improvement was accompanied by a substantial reduction in the proportion of patients meeting the threshold and therefore potentially benefiting from screening with predictive equations.

Our findings are broadly consistent with previous external validation studies showing that currently available predictive equations are unable to reliably replace HCT for determining the need for in-flight oxygen supplementation. However, previous studies were generally limited by relatively small sample sizes, the evaluation of a small number of predictive equations, or inclusion of predominantly COPD populations. In contrast, our study simultaneously evaluated six published predictive equations in a large, heterogeneous cohort that included patients with COPD, ILD, cystic fibrosis, and other respiratory diseases. The consistency of our findings across different disease groups strengthens the evidence that the limitations of these equations reflect fundamental constraints of the prediction models rather than characteristics of a particular patient population.

Comparison with previous studies provides important context for our findings. Billings et al. (Marchand, 2008) retrospectively evaluated 138 patients with chronic respiratory diseases and externally validated the three linear models originally proposed by Dillard et al. (1989) (corresponding to E2–E4 in the present study). Similar to our findings, none of these equations demonstrated sufficient diagnostic performance to replace HCT. However, compared with our cohort, Billings et al. reported substantially higher sensitivities and lower specificities. For example, E2 demonstrated a specificity of only 40% compared with 76% in our study, whereas the sensitivity of E4 declined from 52% in their cohort to only 14% in ours. These differences likely reflect variations in patient populations. The original Dillard equations were derived from only 18 patients with severe COPD (mean FEV_1_ 31 ± 10% predicted) (Dillard et al., 1989) whereas Billings’ cohort had a wider range of diagnoses but only around 12% of subjects with ILD (mean FEV_1_ 52 ± 25% predicted) (Billings et al., 2013). In contrast, our cohort had a substantially greater representation of ILD (36%). These differences in disease severity and case mix may contribute to variable performance of equations across studies. More importantly, these differences illustrate the limited generalizability of predictive equations developed in relatively homogeneous populations when applied to broader clinical cohorts.

Billings et al. subsequently developed seven nonlinear models using the NARMAX approach, with their best-performing model corresponding to E1 in our study. Although this equation demonstrated minimal systematic bias and among the smallest prediction errors in our cohort, its sensitivity for identifying patients requiring supplemental oxygen was only 19%, compared with 64% reported in the derivation study. This marked reduction in performance highlights the importance of external validation and suggests that thresholds optimized within derivation cohorts may not remain valid when applied to independent populations.

E6, also derived by Billings et al., differs from the other models by predicting arterial oxygen saturation rather than PaO_2_. In the original study, saturation-based models consistently performed less well than PaO_2_-based models, a finding that was reproduced in our cohort. Because the oxyhemoglobin dissociation curve is nonlinear, relatively small changes in saturation may correspond to large differences in PaO_2_, particularly within the clinically relevant range near the oxygen supplementation threshold. This physiological limitation likely contributes to the inferior performance of saturation-based prediction models. Unlike the other equations, E6 was also originally evaluated in patients with cystic fibrosis, making it the only model derived from a more heterogeneous respiratory population.

Our findings are also consistent with those of Bradi et al (Martin et al., 2007), who concluded that currently available predictive equations lacked sufficient accuracy for routine clinical use. Although E5 achieved a similar AUC in both studies (0.77 in our cohort versus 0.78 in Bradi et al.), sensitivity and specificity differed considerably. As with the comparisons above, these discrepancies are likely explained by differences in patient characteristics, disease distribution, and the relatively small sample size of the previous validation cohort.

Several additional predictive equations have been published, including those proposed by Muhm (2004) However, these equations were derived from physiological modeling rather than clinical HCT cohorts and were therefore not included in the present analysis. Because our objective was to externally validate equations developed for clinical decision-making using HCT as the reference standard, inclusion of physiologically derived models was considered beyond the scope of this study.

The limitations of normobaric HCT as a surrogate for actual in-flight conditions should also be considered. HCT using 15% FiO_2_ approximates the inspired oxygen tension at the maximum permissible cabin altitude of 8,000 ft, whereas cabin altitude during most commercial flights is frequently lower (Naughton et al., 1995; Dine and Kreider, 2008). Thus, HCT may represent a greater hypoxic exposure than that experienced during many commercial flights. Furthermore, neither HCT nor predictive equations account for flight duration, which can range from short domestic flights to intercontinental flights lasting more than 12 hours. As hypoxia-related symptoms may only become apparent after prolonged exposure (Silverman and Gendreau, 2009), HCT should be considered primarily an assessment of the oxygenation response to hypoxic exposure rather than a comprehensive measure of fitness to fly (Kelly et al., 2008). Therefore, the predicted oxygenation thresholds evaluated in this study should be interpreted in relation to predicted HCT outcomes rather than as absolute thresholds for safe air travel.

The development of a new predictive model was not undertaken in the present study, as our primary objective was to externally validate existing equations in an independent, real-world cohort. Although our findings highlight the need for improved prediction tools, developing a new multivariable model in this relatively heterogeneous cohort could result in overfitting and would require subsequent validation in an independent population. Future studies specifically designed and adequately powered for model development, followed by rigorous external validation, are warranted to determine whether more accurate prediction of in-flight hypoxemia can be achieved.

One of the main strengths of our study is the size and diversity of the study population, representing one of the largest clinical cohorts undergoing HCT reported to date. Unlike previous validation studies, which have largely focused on patients with COPD, our cohort included patients with ILD, cystic fibrosis, and other chronic respiratory diseases, providing a more representative assessment of the performance of predictive equations in routine clinical practice. Furthermore, by externally validating multiple historically important prediction models within a single cohort using standardized HCT as the reference standard, our study provides a comprehensive comparison of their real-world diagnostic performance.

This study also has several limitations. First, its retrospective design introduces the potential for selection bias and limits control over data completeness. Second, arterial blood gases were not routinely obtained during HCT in patients with cystic fibrosis, in whom oxygenation was assessed using SpO_2_ alone. Consequently, PaO_2_-based equations could not be fully evaluated in this subgroup. Nevertheless, this approach reflects contemporary clinical practice, where pulse oximetry is commonly used as a non-invasive surrogate for arterial oxygenation in patients with cystic fibrosis. Finally, a proportion of patients were classified within a heterogeneous “other respiratory diseases” category, limiting disease-specific interpretation within this subgroup.

## 5 Conclusion

In conclusion, none of the six predictive equations evaluated in this study demonstrated sufficient diagnostic performance to safely replace hypoxic challenge testing for determining the need for in-flight oxygen supplementation. Although several equations showed moderate to strong agreement with measured HCT values, this did not translate into reliable classification of patients requiring supplemental oxygen. The principal limitation of the evaluated models was their inadequate sensitivity, resulting in an unacceptable risk of falsely reassuring patients who may develop clinically significant hypoxemia during air travel. Although higher predicted oxygenation thresholds improved negative predictive value for some equations, this was achieved at the cost of excluding most patients from a potential rule-out strategy. These findings highlight the importance of external validation and suggest that predictive equations derived from relatively small, predominantly COPD cohorts do not generalize adequately to broader populations with chronic respiratory disease. While predictive equations may assist in estimating the risk of altitude hypoxemia, HCT should remain the reference standard for evaluating patients in whom the need for in-flight oxygen supplementation is uncertain until more robust prediction models are developed and externally validated.

## Data Availability

The raw data supporting the conclusions of this article will be made available by the authors, without undue reservation.
