# Supplementary material for: External validation of predictive equations for determining the need for in-flight oxygen supplementation in patients with respiratory disease
Source: Front Physiol. 2026 Sep 17;17:1946975. doi: 10.3389/fphys.2026.1946975 (PMC13626894; doi:10.3389/fphys.2026.1946975)
Supplement: Supplementary file 1 [file Table1.pdf]

Supplementary Material

Table 1: Negative predictive value of predictive equations at increasing PaO2 (or SaO2 for E6) thresholds, all diagnoses combined.

Equation	Predicted PaO_2 threshold (mmHg) (or SaO2 % for E6)	% of eligible patients above threshold	NPV (%)
E1	50	90.7	61.5
	55	61.2	75.9
	60	19.4	80.0
	65	4.7	100.0
E2	50	57.4	75.7
	55	20.2	76.9
	60	5.4	100.0
	65	3.1	100.0
E3	50	80.9	63.4
	55	68.7	65.8
	60	52.2	70.0
	65	40.9	76.6
E4	50	89.6	58.3
	55	80.0	63.0
	60	63.5	69.9
	65	52.2	71.7
E5	50	76.7	68.7
	55	52.7	79.4
	60	31.0	77.5
	65	17.8	82.6
E6	85	94.4	61.4
	90	38.3	77.4
	92	7.4	91.7

Table 2: Negative predictive value of predictive equations at increasing PaO2 (or SaO2 for E6) thresholds in COPD patients

Equation	Predicted PaO_2 threshold (mmHg) (or SaO2 % for E6)	% of eligible patients above threshold	NPV (%)
E1	50	88.9	65.6
	55	55.6	80.0
	60	16.7	83.3
	65	2.8	100.0
E2	50	50.0	83.3
	55	16.7	83.3
	60	2.8	100.0
	65	2.8	100.0
E3	50	53.3	81.2
	55	43.3	92.3
	60	30.0	88.9
	65	30.0	88.9
E4	50	70.0	66.7
	55	46.7	85.7
	60	36.7	90.9
	65	30.0	88.9
E5	50	66.7	75.0
	55	41.7	86.7
	60	25.0	77.8
	65	13.9	100.0
E6	85	94.4	61.8
	90	30.6	72.7
	92	5.6	100.0

Table 3: Negative predictive value of predictive equations at increasing PaO2 (or SaO2 for E6) thresholds in ILD patients

Equation	Predicted PaO_2 threshold (mmHg) (or SaO2 % for E6)	% of eligible patients above threshold	NPV (%)
E1	50	91.5	53.7
	55	62.7	73.0
	60	20.3	66.7
	65	3.4	100.0
E2	50	57.6	73.5
	55	20.3	66.7
	60	5.1	100.0
	65	1.7	100.0
E3	50	91.5	55.6
	55	79.7	57.4
	60	66.1	64.1
	65	47.5	71.4
E4	50	100.0	50.8
	55	93.2	54.5
	60	76.3	62.2
	65	62.7	67.6
E5	50	78.0	63.0
	55	55.9	75.8
	60	35.6	71.4
	65	16.9	70.0
E6	85	93.2	54.5
	90	35.6	76.2
	92	3.4	100.0

Table 4: Negative predictive value of predictive equations at increasing SaO2 thresholds in CF patients

Equation	Predicted SaO2 % threshold	% of eligible patients above threshold	NPV (%)
E6	85 %	100.0	66.7
	90 %	39.4	84.6
	92 %	18.2	83.3
